# RECIST criteria: our experience in daily practice

**DOI:** 10.1186/1470-7330-15-S1-P31

**Published:** 2015-10-02

**Authors:** S De Luca, C Carrera, E Casalini Vañek, L Tolkachier, L Alarcon, E Eyheremendy

**Affiliations:** 1 Hospital Aleman, Buenos Aires, Argentina

## Learning objectives

Describe the utility and limitations of RECIST 1.1 criteria.

Emphasise the knowledge of complementary tools for the evaluation of response to treatment of solid tumours.

## Content organisation

Our cases will be presented in a pictorial essay mode. Key differential diagnostic points will be highlighted in the discussion of each case.

It is important to consider:

Complete knowledge of clinical-oncologic status and treatments time points.

Recognition of smallest measured value (NADIR) between time points for adequate selection of comparative review.

Sum of target lesions (baseline and current time points) including percentage of change.

Select no more than two target lesions by sector.

Complete knowledge of actinic extension field which often could present different behavior than other lesional sites.

Local tumour growth over vital organs regardless strict RECIST criteria in exceptional cases.

Further criteria utilization in line with oncological disease being studied (mRECIST criteria for hepatocellular carcinoma or CHOI criteria for GIST).

Use of SUL on baseline PET CT studies with greater accuracy in assessing response on further evaluations (PERCIST criteria).

## Conclusion

RECIST 1.1 proposes internationally accepted criteria to unify and standardise response to treatment of solid tumours in oncologic patients.

These criteria are reproducible but in some oncologic scenarios, we need to widen its use, due to the large number of treatment modalities and possible combined responses.

